# Draft Genome Sequence of a Novel *Chitinophaga* sp. Strain, MD30, Isolated from a Biofilm in an Air Conditioner Condensate Pipe

**DOI:** 10.1128/genomeA.01161-17

**Published:** 2017-10-19

**Authors:** Xuehua Wan, Maxwell Darris, Shaobin Hou, Stuart P. Donachie

**Affiliations:** a Advanced Studies in Genomics, Proteomics and Bioinformatics, University of Hawai‘i at Mānoa, Honolulu, Hawaii, USA; b Department of Microbiology, University of Hawai‘i at Mānoa, Honolulu, Hawaii, USA

## Abstract

Most of the 24 known *Chitinophaga* species were originally isolated from soils. We report the draft genome sequence of a putatively novel *Chitinophaga* sp. from a biofilm in an air conditioner condensate pipe. The genome comprises 7,661,303 bp in one scaffold, 5,694 predicted protein-coding sequences, and a G+C content of 47.6%.

## GENOME ANNOUNCEMENT

As part of a study of the urban microbiome in Honolulu, Hawaii, a heterotrophic bacterial strain designated MD30 was isolated from a biofilm hanging in water flowing from an air conditioning condensate pipe. A comparison of a 1,404-nucleotide (nt) fragment of the MD30 16S rRNA gene with those in the EzBioCloud database revealed the nearest neighbors to be *Chitinophaga niabensis* JS13-10^T^ (1) and *Chitinophaga ginsengisoli* Gsoil 052^T^ (2), both isolated from soil in the Republic of Korea, with 96.5% nucleotide identity over 1,397 and 1,396 nt, respectively. We sequenced the genome of MD30 to provide insights into what may distinguish this putative new *Chitinophaga* species from the type strains of the current 24 species in the genus.

Genomic DNA was isolated from *Chitinophaga* sp. strain MD30 using a phenol-chloroform method, followed by isopropanol precipitation and a 70% ethanol wash. More than 400 Mbp of sequences were generated by various platforms: 93.2 Mbp of shotgun sequences and 152.3 Mbp of 8-kb paired-end sequences by Roche 454 GS FLX+ pyrosequencing; 75.4 Mbp of Illumina paired-end sequences by the Nextera XT DNA library preparation kit and MiSeq v3 sequencing kit; and 85.9 Mbp in 14,254 long reads with an average size of 6 kbp generated by Oxford Nanopore chemistry (R9) and corrected by Canu (3). Different assembly strategies were tested, e.g., Newbler 2.8, Canu, and SPAdes 3.90 (4). Newbler assembled the Roche 454 reads into two scaffolds spanning 7,679,346 bp (*N*_50_ = 7,677,276) but with >160 gaps. SPAdes assembled the 454, Illumina, and Oxford Nanopore sequences, and we improved the assembled scaffolds in npScarf (5). The four scaffolds were compared to the single scaffold assembled by Newbler and then ordered using Mauve (6). After *in silico* gap closing using the BGI GapCloser (http://soap.genomics.org.cn/index.html ) and BWA (7), one scaffold spanning 7,661,303 bp was assembled, with just two gaps remaining. The G+C content of the genome is 47.6%, consistent with the mean of 47.3% ± 3.6% in the genus.

The genome was annotated in the NCBI Prokaryotic Genome Annotation Pipeline (PGAP) (8), Rapid Annotation using Subsystem Technology (RAST) (9, 10), and Prokka 1.11 (11). PGAP identified 5,694 protein-coding genes and 53 tRNA-coding regions. RAST identified 6,082 protein-coding genes and 397 subsystems. Prokka identified 6,029 protein-coding genes and 53 tRNA-coding regions. CRISPRs finder identified one clustered regularly interspaced short palindromic repeat (CRISPR) region with 29 spacers (12, 13). Other than MotB, CheB, CheR, and CheY, most protein components of flagellar and chemotaxis systems were absent. A two-component regulatory system, FixL-FixJ, the heme-based PAS (Per-Arnt-Sim) domain-based oxygen sensor, and its cognate response regulator, were predicted (14). Further genomic analyses will help us understand the role this putatively novel species plays in a biofilm, such as that from which it was cultivated, and also how it differs from other *Chitinophaga* species.

### Accession number(s).

This whole-genome shotgun project has been deposited at DDBJ/EMBL/GenBank under the accession number CP023254. The version described here is the first version.
